# ECCO_2 _removal with a phosphorylcholine-coated membrane oxygenator in difficult respiratory weaning patients

**DOI:** 10.1186/cc12067

**Published:** 2013-03-19

**Authors:** F Turani, S Martini, A Marinelli, M Falco, R Barchetta, F Candidi, F Gargano

**Affiliations:** 1 Aurelia Hospital/European Hospital, Rome, Italy

## Introduction

ECCO_2 _removal may be a useful support in patients with difficult respiratory weaning. The aim of this study is to evaluate a new phosphorylcholine-coated EECO_2 _removal system with no thrombogenic activity to assess the clinical safety of the system, the changes of main cardiorespiratory indices and CO_2 _removal by the system.

## Methods

Ten patients were enrolled. Before starting with ECCO_2 _removal all patients were ventilated with TV <6 ml/kg, peak pressure >35 cmH_2_O and pH <7.25. ECCO_2 _removal was initiated using a modified continuous venovenous hemofiltration system with a membrane oxygenator (ABYLCAP; Bellco, Mirandola, Italy; membrane surface area: 0.67 m^2^, blood flow 280 to 350 ml/minute, phosphorylcholine coated). Femoral vein cannulation with a double-lumen catheter was used to connect the patients to the extracorporeal system. Heparin was infused to maintain ACT <190 to 240 seconds. All patients had ECCO_2 _for 4 days. Every 12 hours the pH, PaCO_2_, peak pressure and PaO_2 _were evaluated. VCO_2 _was determined by indirect calorimetry using a gas analyser equipped on an Engstrom Carestation Ventilator. During the ECCO_2 _removal the patients were ventilated with TV ≤6 ml/kg and peak pressure <30 cmH_2_O. All data are expressed as mean ± SD. One-way ANOVA was used to compare the changes of parameters. *P <*0.05 was considered significant.

## Results

Table 1 presents the main results. The CO_2 _removal by membrane oxygenator ranged from 56 to 37 ml/minute. All patients survived to the treatment and 7/10 were weaned from the ventilator at the end of ECCO_2 _removal. Only one oxygenator was used for every patient without clotting of the circuit or any major bleeding problem.

**Table 1:** 

	Day 0	Day 2	Day 4
pH	7.24 ± 0.06	7.38 ± 0.1	7.41 ± 0.07*
PaCO_2 _(mmHg)	70 ± 5	57 ± 8	52 ± 3*
PP (cmH_2_O)	48 ± 4	28 ± 4*	25 ± 4
VCO_2 _(ml/minute)	220 ± 15	201 ± 14	180 ± 13*

*P <0.05 versus T0.

## Conclusion

ECCO_2 _removal with a phosphorylcholine-coated membrane oxygenator is clinically safe, avoids clotting of the oxygenator circuit and allows adequate CO_2 _removal.
